# Chromosome-Scale Assembly of Novel *Caenorhabditis sp. 65* (strain JU4118)

**DOI:** 10.17912/micropub.biology.002448

**Published:** 2026-10-04

**Authors:** Jasbelle Sosa, Michelle A. McCauley, Victoria K. Eggers, Janna L. Fierst, Karolina Willicott

**Affiliations:** 1 Biological Sciences, Florida International University, Miami, FL, United States

## Abstract

We present a chromosome-scale assembly of strain JU4118, an inbred line of *Caenorhabditis* *sp. 65* . This species is a wild isolate that was sampled in Da Lat, Vietnam by the Marie-Anne Félix lab in December 2019. This strain has a genome size of 126 Mb, with a GC content of 37.95 %. Comparisons of *C. sp. 65* with *C. elegans* show conserved broad-scale chromosomal organization, but finer-scale variation which may be clade-specific, such as expansion of the X chromosome and increased repeat content. By assembling and annotating its genome, we aim to expand future evolutionary studies on the *Caenorhabditis* genus.

**Figure 1. PacBio and Hi-C sequencing leads to a chromosome-scale assembly of novel Caenorhabditis sp. 65:** **A.** Snail plot (BlobTk) of assembly statistics for JU4118. The outer circumference of the plot represents the full length of the genome. The rings, from outer- to inner-most describe the features: the blue rings represent %GC content per scaffold; the pale orange ring represents N90 length in Mb; the bold orange ring represents N50 length in Mb; the red wedge is the longest scaffold length in Mb; the dark gray represents the length of each scaffold, arranged from longest to shortest in a clockwise direction; the light gray represents total scaffold count on a logarithmic scale. The top right circle describes BUSCO scores, displaying a summary of complete, duplicated, fragmented, and missing genes in the nematoda_odb12 set. **B.** Hi-C contact map of JU4118 assembly reveals the six chromosome-scale scaffolds (X sex chromosome, autosomes I-V, ordered from largest to smallest. Scaffolds 7-10 are unplaced **C.** Synteny plot of single-copy orthologs from pairwise comparisons of *C. sp. 65* , *C. elegans* , and *C. doughertyi* . Colors correspond to *C. elegans* chromosomes. **D.** *Caenorhabditis* phylogeny of Elegans Group, with *C. japonica* as the outgroup species. **E.** Percent of nucleotides annotated as genes (red), repeats (blue), or GC content (green) in 10 kb bins across each chromosome **. F.** Chromosomes with 100 kb binned counts of single-copy orthologs to *C. elegans* genes previously assigned to Nigon elements (A-E, N, X). **G.** Kimura-2 parameter distance showing TE "age" of the major TE subclasses annotated in JU4118. **Table 1.** Properties of *Caenorhabditis sp. 65*

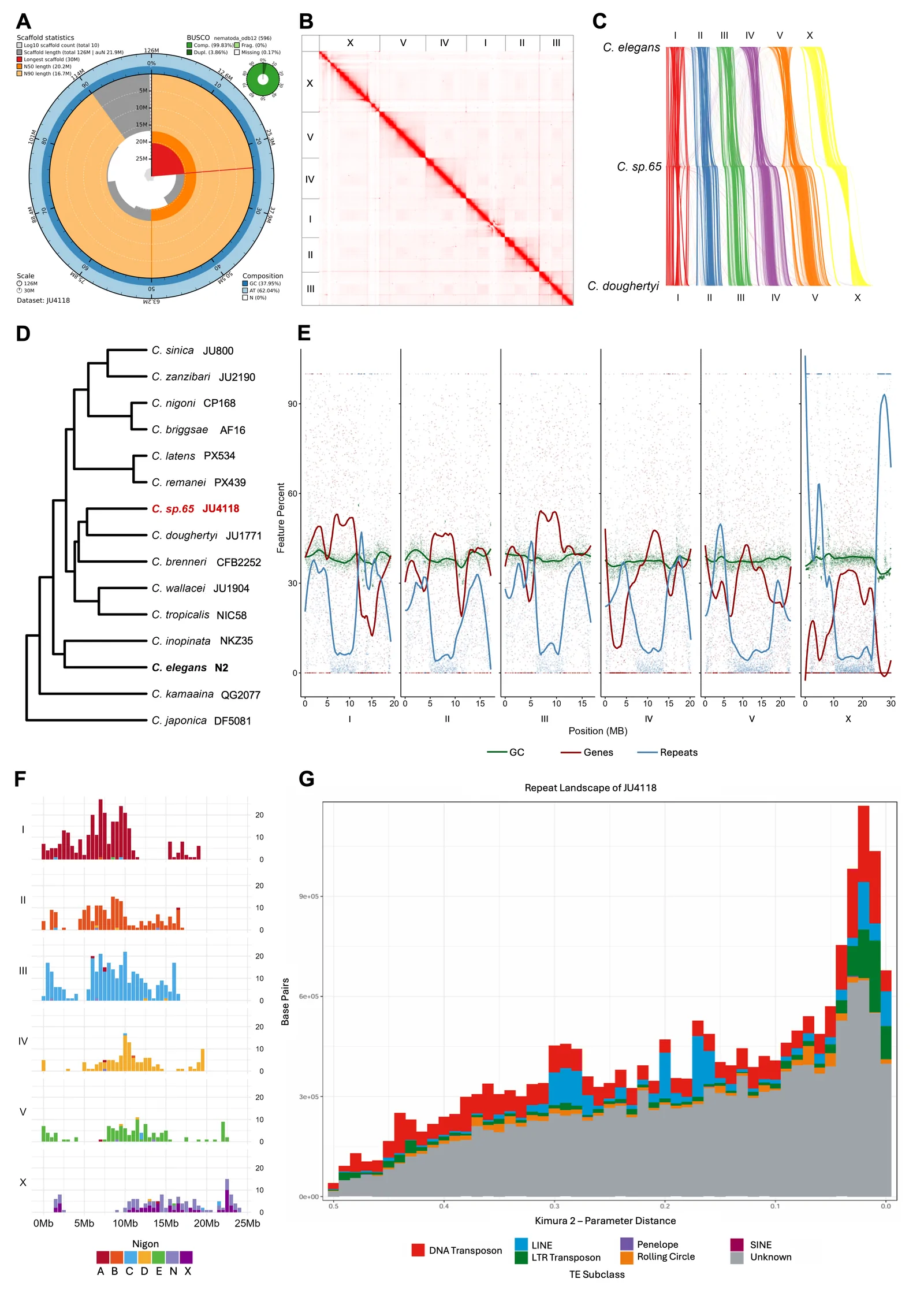



**Table:** 

Property	Metric
Location found	Da Lat, Vietnam
Location coordinates	12.17523, 108.69884
Mating system	Dioecious
Genome size (Mb)	126
Approximate coverage (x) – PacBio HiFi	171
Approximate coverage (x) – Illumina Hi-C	643
Number of reads (Gb) – PacBio HiFi	21.6
Number of reads (Gb) – Illumina Hi-C	81.3
GC content (%)	37.96
Number of protein coding genes	18,221
Repeats (%) – RepeatMasker	20.11
Repeats (%) – EarlGrey	24.6
SRA accession no. – PacBio HiFi	SRR38757051
SRA accession no. – Illumina Hi-C	SRR38757052
Isolated by	Marie-Anne Félix lab

## Description

*Caenorhabditis* is a genus of the phylum of Nematoda that consists of diverse roundworms, including the species *Caenorhabditis elegans* . *C. elegans* has become an important model organism in biology. In an effort to understand further evolutionary biology of *Caenorhabditis* , many species have been sampled around the world to be sequenced and described (Stevens et al., 2019). Here, we aim to describe *Caenorhabditis* *sp. 65* (strain JU4118). Morphologically, *C. sp. 65* is similar in size and appearance to *C. elegans* when viewed under a stereo-microscope. The mating systems of the two species, though, differ. *C. sp. 65* is a dioecious species, whereas *C. elegans* is androdioecious. *C.* *sp. 65* was isolated from rotting fruit in Da Lat, Vietnam by the Marie-Anne Félix lab (Table 1) and was inbred for 25 generations. Phylogenetic analysis places *C. sp. 65* as a sister to *C. doughertyi* , an outcrossing species with a genome size of ~147 Mb ( Figure 1, Panel D).

Using PacBio HiFi long-read sequencing (~171× coverage) and Hi-C Illumina paired-end sequencing (~643× coverage), assembly with Hifiasm (Cheng et al., 2021) resulted in 471 contigs spanning ~203 Mb. After removal of bacterial contigs using BLAST (Camacho et al., 2009), purging haplotigs with purge_dups (Guan et al., 2020), and scaffolding with YaHS (Zhou et al., 2023), the final genome assembly was 126 Mb in 10 scaffolds ( Figure 1, Panel A). Scaffolds were assigned chromosomal identities based on synteny with *C. elegans* , revealing five autosomes, the X chromosome, and four smaller unplaced scaffolds ( Figure 1, Panel B). Nucleotide BLAST (Camacho et al., 2009) reveals that three of the four unplaced scaffolds have similarity to several known *Caenorhabditis* species. The other unplaced scaffold contains about 98% repeats and does not have similarity to any known sequences.

The chromosome-level assembly of *C. sp. 65* is highly syntenic with *C. elegans* and shows similar patterns of chromosomal organization, consistent with other complete genomes in the Elegans Group. Identification of Nigon elements, ancestral linkage groups that show macro-syntenic changes resulting in present-day karyotypes, reveals the same pattern as that seen across *Caenorhabditis* : nigons A, B, C, D, and E correspond to chromosomes I, II, III, IV, and V, respectively, while the X is a fusion between nigons N and X ( Figure 1, Panel E) (Tandonnet et al., 2019). Across all chromosomes, repeat sequences generally appear higher in density on the first and last quarter of the chromosome, often referred to as the chromosome “arms.” Appearance of genes displays the opposite pattern: genes are more frequent in chromosome centers ( Figure 1, Panel F). GC content also has a similar landscape across the chromosomes as compared to *C. elegans* , hovering around 38% across the genome (Correa et al., 2025).

Despite the conservation of macrosynteny, within chromosomes there are several noticeable rearrangements, inversions, and expansions ( Figure 1, Panel C). For example, *C. sp. 65* shows an expansion on the X chromosome relative to *C. elegans .* This enlarged X chromosome, and the enlarged genome in general, seems to be a feature of this clade as it is also present in *C. doughertyi* and *C. sp. 61* (Lad et al., 2026). The genome sizes of *C. elegans* , *C. sp. 65* , *C. sp. 61* , and *C. doughertyi* are 100 Mb, 126 Mb, 165 Mb, and 147 Mb, respectively. The X chromosome lengths are 18 Mb, 30 Mb, 42 Mb, and 32 Mb, respectively. Dioecious species are known to have larger genome sizes than androdioecious species, and several theories exist as to why this might be, including: gene family turnover (Adams et al., 2023), accumulation of repetitive elements (Woodruff and Teterina, 2020), and intron size variation (Stevens et al., 2019).

The *C. sp. 65* predicted gene set had a BUSCO (Manni et al., 2021) completeness of 99.5%. The assembly contained 18,221 predicted genes, making up 31.5% of the genome. This is slightly less than *C. elegans* , which has approximately 20,000 genes. Accordingly, there were more *C. elegans* -specific and *C. sp. 65* -reduced orthogroups than *C. sp. 65* -specific and *C. sp. 65* -expanded orthogroups. GO term analysis of OrthoFinder (Emms & Kelly, 2019) output shows that *C. sp. 65* -specific genes are enriched in processes underlying embryo development, negative regulation of vulval development, innate immune response, and ubiquitin-dependent protein degradation. *C. elegans* -specific genes are enriched in processes related to sensing the environment, including GPCR signaling pathways, sensory perception of a chemical stimulus, and olfactory behavior. However, many genes were lacking in functional annotation. For example, the region of expansion on the X chromosome showed many genes with no functional domains annotated, and those that were, tended to be predicted disordered regions.

The *C. sp. 65* assembly was annotated by EarlGrey (Baril et al., 2024) to be composed of 24.6 % repetitive elements, slightly more than *C. elegans* 20.5 %. Percent repeats for each chromosome of *C. sp. 65* was: 25.5 %, 19.2 %, 24.9 %, 22.5 %, 18.8 %, and 32.7 %, in order of chromosome I, II, III, IV, V, and X. Similarly, the percent repeats across *C. elegans* was: 23.4 %, 21.2 %, 24.8 %, 19.1 %, 21.6 %, and 14.1 %. The X chromosome of *C. sp. 65* had a percentage of repeats twice that of *C. elegans* . The same pattern has been seen in *C. sp. 61* (Lad et al., 2026). Repeat classifications showed more long interspersed nuclear elements (LINEs) (1.3 % vs 0.6 %), long terminal repeats (LTRs) (1.0 % vs 0.4 %), simple repeats (8.0 % vs 4.6 %), and unclassified repeats (10.6 % vs 4.0 %) in *C. sp. 65* compared to *C. elegans* . Kimura distance (Kimura, 1980) which is often used as a proxy for transposable element “age” but simply a measure of sequence divergence, reveals periodic small bursts of LINEs, and a recent expansion of LINEs, LTRs, DNA transposons, and unclassified repeats ( Figure 1, Panel G).

Telomeric-repeat motifs (TRMs) can also be found in this assembly. Telomeres are composed of tandem arrays of TRMs that are recognized by telomere-binding proteins that ensure replication and protection of chromosomal ends. TRMs are conserved in Nematoda, with the canonical TRM sequence of TTAGGC (Lim et al., 2023). We found that all chromosomes, except I and V, in the JU4118 assembly contain the canonical TRM at the 5' terminus. At the 3' terminus, all chromosomes, except II, contain the canonical sequence. The unplaced scaffolds do not appear to have TRM sequences at either terminus.

More work is needed to understand the evolutionary forces shaping *Caenorhabditis* genomes, both within this clade and across the genus in general. Our draft genome of *C. sp. 65* , JU4118 provides a resource for these future molecular, comparative, and phylogenetic evolutionary studies.

## Methods

**Collection** : Worms were kept in cryopreservation until transfer to our research group and were maintained continuously thereafter using standard *Caenorhabditis elegans* techniques. Worms were grown on agar plates at 20°C using nematode growth media and seeded with a lawn of *Escherichia coli* strain OP50 *.* For DNA extractions, worm populations were expanded by transferring a small “chunk” of agar to three 100 mm plates seeded with *E. coli* and left at 20°C for 2-3 days until plate was filled with mixed-age worms but free of dauer larvae. Worms were then washed off plates with M9 buffer into a 15 mL conical tube and washed twice with M9 buffer to minimize surface contaminants, then resuspended in 10 mL M9 and left on a rocker overnight (~17 hours) to purge gut of further contaminants. Prior to extraction, worms were washed twice more with M9. Worms were collected by pelleting worm bodies via centrifugation, removing M9 supernatant, and transferring 50 µL aliquots of worm pellet to 1.5 mL tubes.

**Long-read sequencing:** DNA for long-read sequencing was extracted using the Promega Wizard® HMW DNA Extraction Kit (cat. no. A2920), using the manufacturer protocol with minor modifications. Worm cuticles were broken by repeated freeze/thaw cycles where one tube of live worm pellet was placed at −80°C for five minutes, moved to 37°C until thawed, briefly vortexed, and then frozen again, for five cycles. All centrifuge steps were done at 4°C and alcohols were kept on ice until use. At the lysis step, an extra incubation step of 25 minutes at 65°C was added. PacBio sequencing was performed at the University of Miami's John P. Hussman Institute for Human Genomics Sequencing Core Facility (RRID:SCR_017828).

**Hi-C:** Extra tubes of 50 µL worm pellet were frozen at −80°C using a Mr. Frosty™ freezing container (Thermo Scientific cat. no. 5100-001) filled with 100% isopropanol to prevent ice crystal formation. Two tubes were then mailed on dry ice to Arima Genomics for High Coverage Chromatin Conformation Capture sequencing (Hi-C).

**Genome Assembly:** PacBio HiFi and Arima Hi-C libraries were assembled using Hifiasm v0.16.0 (Cheng et al., 2021) using default parameters. BLAST v2.14.1 (Camacho et al., 2009) was used to identify and remove contaminant contigs from the diploid and phased haploid assemblies. PacBio HiFi reads were mapped to the assembly using minimap2 v2.30 (Li, 2018), with parameter -x map-hifi and read depth cutoffs were calculated with pbcstat from purge dups v1.2.6 (Guan et al, 2020). Assembly self-alignment was performed with minimap2 v2.30 and parameters -xasm5 -DP to find duplications. Alternative haplotypes were subsequently removed with purge_dups v1.2.6. To quality check the assemblies between each step, QUAST v5.3.0 (Gurevich et al., 2013) was used with default parameters and BUSCO v6.0.0 (Manni et al., 2021) was run against lineage dataset Nematoda odb12 with option -m genome and --offline.

**Phylogenetic Analysis:** A multigene tree was calculated from the BUSCO output of 95 species within Rhabditidae. Single copy orthologs were concatenated for multiple sequence alignment, which was completed with MAFFT v.7.221 using the localpair option with 1000 times iterative refinement (Katoh and Standley, 2013). Alignments were trimmed using ClipKIT v.2.3.0 with option smart-gap (Steenwyk et al., 2020) and partitioned with AMAS concat (Borowiec, 2016). IQ-TREE v.1.6.12 was used to compute the phylogenetic tree with option -m MFP (Nguyen et al., 2015). Branch length supports were calculated with SH-like approximate likelihood ratio test and 1000 bootstrap replicates (Guindon et al., 2010). Visualization was done in R with package phytools (Revell, 2012). Tips containing species too distant from *C. sp. 65* were dropped for clarity.

**Hi-C Mapping:** Juicer v2.0 (Durand et al., 2016) was used for alignment and processing of Hi-C raw data using default parameters, with the --assembly option. YaHS v1.2.2 (Zhou et al., 2023) was used for scaffolding. Juicebox v2.3.6 (Robinson et al., 2018) was used for visualization and assessment.

**Gene and Repeat Annotation:** Gene annotation was performed with BRAKER3 v3.0.8 (Gabriel et al., 2024) on the softmasked assemblies using the protein dataset Nematoda odb10 and RNA sequence data downloaded from NCBI project PRJNA1256413 (O'Leary et al., 2024). Prior to BRAKER3, genomes were softmasked with RepeatModeler2 (Flynn et al., 2020) and RepeatMasker (Smit et al., 2013), and RNA reads were aligned to the genome with STAR v2.6.1a and option --outSAMstrandField intronMotif (Dobin et al., 2013). Briefly, BRAKER relies on 2 generalized hidden markov models for gene prediction, GeneMark (unsupervised) and AUGUSTUS (supervised) (Brůna et al., 2024; Stanke et al., 2006). The resulting protein sets are then combined by TSEBRA (Gabriel et al., 2021) to maximize BUSCO completeness scores. Protein predictions were then filtered for the longest isoform using AGAT v1.4.1 (Dainat et al., 2026), specifically the scripts agat_sp_keep_longest_isoform.pl and agat_sp_extract_sequences.pl. Statistics were generated with agat_sp_statistics.pl. Functional annotations were done with InterproScan v5.68.100.0 with options -dp -goterms -pathways (Jones et al., 2014). OrthoFinder v2.5.5 (Emms & Kelly, 2019) was used to find single copy orthologs between JU4118 and *C. elegans .* The six major chromosomes were identified by location of single copy orthologs on *C. elegans* chromosomes *.* Nigon element classifications were assigned to single copy orthologs using a list of known gene:Nigon associations from Gonzalez de la Rosa (2021). Finally, GO term enrichment analysis was conducted in R with package topGO (Alexa & Rahnenführer, 2026).

Repetitive elements were annotated with EarlGrey v6.0.1 (Baril et al., 2024) and options -r nematoda -e yes. Briefly, EarlGrey employs a BLAST, Extract, Align, Trim (BEAT) process adapted from TEStainer (https://github.com/jamesdgalbraith/TEstrainer) along with subprocesses: Tandem Repeat Finder (Benson, 1999), MREPS (Kolpakov et al., 2003) SA-SSR (Pickett et al., 2013), LTR_FINDER (Xu & Wang, 2007), RepeatModeler2, and RepeatMasker. EarlGrey merges and defragments the annotations with RepeatCraft (Wong & Simakov, 2019) to produce the final consensus library.

**Snail Plots** : Snail plots were generated by BlobTk v0.8.0 (Challis & Blaxter, 2026) using the soft-masked assemblies. Assembly BUSCO scores were recalculated for the snail plots using BUSCO v6.0.0 (Manni et al., 2021) and the nematoda_odb12 dataset.

**Data availability**

Bioinformatic scripts, workflows and software commands are available at https://github.com/jannafierst/HiC_Assemblies

Scaffolded genome and annotation supplementary files are available at https://zenodo.org/records/21998323

**Nucleotide sequence accession numbers**

DNA libraries used in this project have been deposited at the Sequence Read Archive (SRA): SRR38757051 (PacBio) and SRR38757052 (Hi-C) under NCBI under Bioproject PRJNA1256413 .
